# Toxicity of Cadmium, Copper, and Zinc to the Threatened Chiricahua Leopard Frog (*Lithobates* [*Rana*] *chiricahuensis*)

**DOI:** 10.1007/s00128-017-2188-1

**Published:** 2017-11-02

**Authors:** Robin D. Calfee, Edward E. Little

**Affiliations:** 0000000121546924 grid.2865.9 U.S. Geological Survey, Columbia Environmental Research Center, 4200 New Haven Road, Columbia, MO 65202 USA

**Keywords:** Leopard frogs, Acute and chronic exposure, Copper, Cadmium, Zinc, Metals

## Abstract

The Chiricahua leopard frog (*Lithobates chiricahuensis*) is in decline throughout the western United States, and is particularly sensitive to physical, chemical and biotic changes in their habitat. Acute toxicity tests revealed that among the metals detected in Chiricahua leopard frog habitat, copper was toxic at concentrations lower than those observed in the environment. Developing tadpoles were chronically exposed for 60 days to cadmium, copper and zinc because of the potential for long term exposure to these metals during early development. Cadmium was toxic, but at concentrations above observed environmental levels. Copper was especially toxic to this species at concentrations of about 10% of concentrations observed in their habitats. The onset of toxicity occurred within a few days of exposure, thus pulsed exposures from rain events could potentially be acutely toxic to tadpoles of this species. Zinc did not appear to have a negative impact during the acute or chronic exposures.

Many frog species within the family Ranidae, particularly in the southwestern United States have experienced notable population declines across their ranges (Hale and Jarchow 1988; Clarkson and Rorabaugh 1989; Sredl 1997). The Chiricahua leopard frog (*Lithobates* [*Rana*] *chiricahuensis*) has disappeared from entire mountain ranges and river drainages and often exists in isolated populations because of habitat fragmentation and the loss of aquatic corridors (Sredl and Howland 1995). It was federally listed as a threatened species in 2002 (U.S. Fish and Wildlife Service 2002). Many factors have been suggested as potential causes for global amphibian decline such as climate change, increases in UV-B radiation exposure, disease, and contaminants. The Chiricahua leopard frog (hereafter, CLF) reproduces in springs, rivers, ponds, lakes, and livestock tanks in arid, mountainous habitat and has a larval period of 3–9 months. Due to the complex life-cycle, each stage of development during the frog’s life history has specific environmental requirements thus rendering it particularly sensitive to physical, chemical and biotic changes in their aquatic habitat (U.S. Fish and Wildlife Service 2002). There is a paucity of information about environmental contamination in CLF habitats. Chemical pollution of aquatic breeding sites (especially by certain metals from mining and smelting activities) is one of the potential causes for the decline of the CLF (Hale and Jarchow 1988, U.S. Fish and Wildlife Service 2002). Metals and other contaminants occur in water bodies due to aerial deposition, upstream contamination, and leaching from bedrock. Metals, particularly cadmium, copper, and zinc have been detected in water and in amphibian tissues collected downstream and downwind of smelters. Mining for copper, gold, iron, and other minerals was historically prevalent within CLF habitats, and although current mining activity is limited to only a few localities (i.e. California Gulch, Pajarito Mountains, Arizona), historic and current mining activities still pose a potential threat to the CLF (U.S. Fish and Wildlife Service 2002). The objectives of the study were to determine the sensitivity of CLF to metals that are often detected in their habitat, including cadmium and copper in 96 h acute exposures and cadmium, copper, and zinc in 60 day chronic exposures. Data generated by these studies may provide toxicological reference for the evaluation of concentrations observed in the field.

## Materials and Methods

Portions of several CLF egg masses were collected in July 2005 and April 2007 from a pond on private property in southwestern New Mexico (Catron County). The man-made pond is fishless, permanent, and fed by a small creek that drains from upland Ponderosa pine (*Pinus ponderosa*) habitat owned by the U.S. Forest Service. Environmental parameters measured in the pond between 1200 and 1300 h. in 2007 were: water temperature, 25°C; dissolved oxygen, 13.5 mg/L; pH, 9.66; hardness, 121 mg CaCO_2_/L; and the air temperature was 31°C. Each egg mass was placed in a separate 3.8-L plastic freezer bag partially filled with pond water. The bags were sealed and placed in plastic coolers with blue ice that were transported on the following day to the U.S. Geological Survey, Columbia Environmental Research Center (CERC, Columbia, MO). The egg masses were slowly acclimated from 18 to 22°C then placed in two separate 37.8-L glass aquaria located in a water bath maintained at 22 ± 1°C. The eggs rested on plastic grating to reduce the chance of hypoxia. The water in the aquaria was an aerated 50:50 mixture of well water and deionized water. Once the tadpoles hatched, they were fed algae discs (Wardley^®^ Premium Algae Discs^™^, Secaucus, NJ) and ground fish food flakes (Tetra Min^®^ Tropical Flakes, Blacksburg, VA). Partial water changes (about 25% water replacement) with blended water were made every other day for five days after hatching. Thereafter, 100% water changes were made and aquaria were hand-scrubbed every 6 days. The tadpole diet was eventually replaced by gelatin cubes of crushed algae discs, fish flakes, minced fresh cucumber, and calcium powder (Rep-Cal^®^ Phosphorus-Free Calcium with Vit.D_3_, Ultrafine Powder, Los Gatos, CA). The collection and husbandry of CLF was conducted in accordance with protocols developed forArizona leopard frogs (U.S. Fish and Service 2006). The tadpoles were staged following the tables in Gosner (1960).

The metal salts were obtained from Sigma-Aldrich (St. Louis, MO). A stock solution of each metal (copper II sulfate pentahydrate, cadmium chloride hemi-pentahydrate, and zinc chloride) was prepared by adding the ACS reagent grade (98% purity) metals to deionized water. Test stock solutions were prepared 24 h before the start of exposures in volumetric flasks and wrapped with aluminum foil to reduce exposure to ambient light and placed in the refrigerator. At the initiation of an exposure, spike amounts of each stock solution were added to the test chambers to acquire the desired concentration series.

In range-finding tests, static acute toxicity tests were conducted with early (Gosner stage 25) and late (Gosner stages 28–31) tadpole life stages following ASTM guidelines (ASTM 2002). The tadpoles were exposed to copper and cadmium for 96 h to determine the lethal concentration to 50% of the test population (LC50). The experimental units were 3.8-L, glass, wide-mouth jars filled with 2-L of a 50:50 mixture of well water to deionized water. The tadpoles were exposed at 22°C to five metal treatments (50% serial dilutions) and a control with three replicates for each treatment. Tadpoles were not fed during the 96 h exposure. The number of tadpoles tested, number of replicates and loading mass of tadpoles in the 96 h exposure were in accordance with ASTM (2002) guidelines.

Chronic toxicity tests were conducted for 60 days following ASTM guidelines (ASTM 2002). The exposures to cadmium (0 to 351 µg/L), copper (0 to 165 µg/L) and zinc (0 to 165 µg/L) were initiated with Stage 25 tadpoles under static-renewal conditions. There were three replicate treatments per concentration with three tadpoles per replicate. All tests were conducted in a 50:50 mixture of well water to deionized water having a water hardness and alkalinity of about 100 mg/L as CaCO_3_. The test solutions were renewed twice weekly by replacing about 95% of the water with fresh test solutions throughout the 60 days exposure. The tadpoles were fed 12 h before each water change. Test jars were randomly arranged within a single block in a water bath maintained at 22 ± 1°C. Daily checks were made for mortality and any dead tadpoles were removed. At the end of the 60 days exposure, weight, length and Gosner stage was recorded for each surviving individual tadpole.

The temperature of the water bath was monitored by placing maximum/minimum sensors (Fisher Scientific Traceable^®^ digital thermometer) in the center and at both ends of the experimental array. Test water was measured for dissolved oxygen, pH, alkalinity, hardness, and conductivity once a week. Dissolved oxygen (mg/L) was measured with an YSI Model 54A oxygen meter and YSI 5739 probe (YSI, Yellow Springs, Ohio). Conductivity (µS/cm) was measured with an Orion 140 S–C–T Meter and a 014010 conductivity cell (Thermo Scientific, Waltham, Massachusetts). Alkalinity (mg/L CaCO_3_) and pH were measured with an Orion EA940 Expandable Ionalyzer, Orion 917001 ATC probe, and Orion 8165BN combination pH probe (Thermo Scientific). Total hardness was measured by complexometric titration (ethylenediaminetetraacetic acid [EDTA]). Total ammonia was measured with an Orion EA940 Expandable Ionalyzer and Orion 95–12 ammonia electrode (Thermo Scientific). Dissolved oxygen and ammonia concentrations were monitored twice per week to ensure sufficient water quality during the exposures. Water samples were taken for metals analysis at 30 and 60 days of exposure.

To sample for metals analysis, 24 mL of test water was drawn into a polypropylene syringe to which an acid-rinsed, Teflon “sipper straw” was attached. The sipper straw was removed from the syringe and a polypropylene filter cartridge housing a 0.45-µm pore size, polyethersulfone membrane was attached. About 4 mL of sample was dispensed through the filter to waste, followed by 20 mL of sample that was collected in an acid-cleaned, polyethylene bottle. Each sample was acidified to 1 percent (v/v) with high-purity, 16M nitric acid and stored for as many as 3 months before analysis was performed by inductively-coupled plasma mass spectrometry (Brumbaugh et al. 2007). For cadmium, recovery was 101.25% (± 0.96 standard deviation) of spiked sample, blank concentration was 0.5 (< 0.05 µg/L), reference concentration was 23 µg/L (± 0.9), and duplicate deviation was 1.2% (± 1.3 SD). For copper, recovery was 97.6% (± 2.2) of spiked sample, blank concentration of copper in was 0.002(± < 0.002) µg/L, reference concentration was 0.084 (± 0.084) µg/L, and duplication among samples was 14% (± 29.49). For zinc, recovery was 97.3% (± 1.35) µg/L of spiked sample, blank concentration was 0.003 µg/L (± 0.003) µg/L. Reference sample concentration was 0.0543 (± 0.054) µg/L, and duplication among samples was 1.56% (± 3.49).

Acute-to-Chronic Estimation (ACE) with time-concentration–effect models (USEPA 2003) and Accelerated Life Testing procedures (Mayer et al. 2002; Sun et al. 1995) were used to calculate LC_50_ values and 95 percent confidence intervals for each metal based on observed concentrations measured during the chronic exposure. In the ACE software, the model is applied to organisms exposed under acute conditions and the variable measured is time to death. The model assumes that both exposure concentration and duration affect survival probability, and thus has the ability to summarize the entire concentration-time-response data of the toxicity test. The criterion of non-overlapping 95 percent confidence intervals was used to determine significant differences (*p* < 0.05) among treatment concentrations (APHA 1971). Separate analyses were conducted for each chemical that caused ≥ 40% mortality in at least one concentration in order to calculate the LC50. When the existence of null data (no mortality) prevented computation, a total control mortality of “0” was changed to “1”. A Least Significant Means test was conducted to determine if mortality, growth, and development of CLF exposed to metals were significantly different from the controls.

## Results and Discussion

Acute tests revealed that among the metals that have been detected in CLF habitat, copper appeared to be particularly toxic with 100% mortality in the highest test concentration of 0.5 mg/L for the static acute exposures (Table 1). The 96 h LC50 observed during the static acute tests ranged from 0.22 mg/L for Gosner Stage 25 tadpoles to 0.34 mg/L for more developed Gosner 28–31 stage tadpoles. These values are well below the maximum observed environmental concentration (47.5 mg/L) for copper (Table 1). Cadmium was also toxic however the LC50 values of 13.8 mg/L for both early and 15.0 mg/L for late stage tadpoles were well above the maximum observed environmental concentration (5.0 mg/L). Because of the possibility for long term exposure, 60-day chronic exposures were conducted. These tests included exposures to cadmium, copper, and zinc, individually.

**Table 1:** Test concentration series for each metal used in 96-hour acute exposures along with the maximum observed environmental concentration (OEC; Wiemeyer et al. 2004) and estimated LC50 (lethal concentration affecting 50 percent of test population) for early and late stage tadpoles with 95% confidence intervals in parentheses

Concentration series (mg/L)	Metal			
	Copper		Cadmium	
	Early stage (Gosner 25)	Late stage (Gosner 28–31)	Early stage (Gosner 25)	Late stage (Gosner 28–31)
Control	0.016		0.47	
Low	0.031		0.94	
Med-Low	0.063		1.88	
Medium	0.125		3.75	
Med-Hi	0.25		7.5	
High	0.5		15	
LC50 (mg/L)	0.22 (0.18–0.26)	0.34 (0.27–0.41)	13.8 (9.70–17.9)	> 15
OEC^*^	47.5 mg/L		5.0 mg/L	

*Observed environmental concentration

Water quality variables (dissolved oxygen, pH, alkalinity, hardness, conductivity and temperature) observed during exposures to metals remained within acceptable limits throughout the tests. The concentration of metals applied during the 60-day exposure is summarized in Table 2. On day 30 of the exposure the high concentration samples were taken due to mortality occurring in the high treatment whereas the low concentration samples were taken on day 60 of exposure to monitor the chronic response observed. Survival among CLF tadpoles exposed to control conditions was 100% during exposures to each metal.

**Table 2:** The average measured concentration of cadmium, copper, and zinc (standard deviation in parentheses, N = 18) during the 60 day exposure

Metal	Average concentration (µg/L)
Cadmium	Blank < 0.5
	Control < 0.5
Low	0.7 (0.1)^a^
	3.0 (0.5)
Medium	19.3 (2.2)
	110.8 (8.6)
High	351.5 (10.6)^b^
Copper	Blank < 0.2
	Control < 0.2
Low	0.8
	3.0 (2.0)^a^
Medium	6.8 (2.2)
	46.8 (14.2)
High	165.0 (7.1)^b^
Zinc	Blank < 0.3
	Control < 0.3
Low	0.8 (3.0)^a^
	4.0 (1.2)
Medium	10.6 (1.2)
	62.0 (5.1)
High	165.0 (7.1)^b^

^a^Measured day 60

^b^Measured day 30

The LOEC concentrations for cadmium determined during the 60-day chronic exposures were 111 µg/L for survival and length (Tables 3, 4). Exposure to 19 µg/L significantly enhanced growth compared to controls. (Table 4). Because environmental concentrations of cadmium may reach 5 mg/L (R. MacRae personal communication) injury to CLF may result from chronic exposures. A review of published cadmium studies with amphibians reveals toxicities ranging from 0.009 to 8.18 mg/L (Linder and Grillitsch 2000), with an LC50 of 3.7 mg/L reported for both *Rana pipiens* and *Rana catesbeiana* tadpoles (Zettergren et al. 1991). Our results indicate that cadmium is much more toxic under chronic exposure conditions than during short-term exposures.

**Table 3:** Mean cumulative mortality observed during a 60 day exposure of Chiricahua leopard frog tadpoles to cadmium, copper, or zinc

Measured metal concentration µg/L	Percent mortality			
	2 weeks	4 weeks	6 weeks	8 weeks
Cadmium				
Control	0	0	0	0
0.7	0	0	0	0
3.0	0	0	0	0
19.3	0	0	0	0
110.7	0	11 (0.33)	11 (0.33)	11 (0.33)
351.5	11 (0.33)	11 (0.33)	11 (0.33)	11 (0.33)
Copper				
Control	0	0	0	0
<2.0	0	0	0	0
3.0	0	0	0	0
7.0	0	0	0	0
46.5	11 (0.33)	11 (0.33)	22 (0.44)	22 (0.44)
165.0	100	100	100	100
Zinc				
Control	0	0	0	0
< 3.0	0	0	0	0
4.0	0	0	0	0
10.6	0	0	0	0
62.0	0	0	0	0
165.0	0	0	0	0

Standard deviation shown in parentheses. N = 18 per metal

**Table 4:** Average weight, length, and developmental stage of Chiricahua leopard frog tadpoles following a 60 day exposure to cadmium, copper, or zinc (standard deviation in parentheses)

Measured metal concentration μg/L	Growth parameters		
	Length (cm)	Weight (mg)	Gosner stage
Cadmium			
Control	3.14 (0.21)	292.2 (0.07)	29 (0.81)
0.7	3.28 (0.26)	276.7 (0.08)	29 (0.99)
3.0	3.24 (0.21)	293.3 (0.07)	28 (0.81)
19.3	3.52 (0.21)	398.9* (0.07)	30* (0.81)
110.7	2.84* (0.24)	235.7 (0.07)	27 (0.92)
351.5	–	–	–
Copper			
Control	3.33 (0.21)	347.8 (0.07)	29 (0.81)
< 2.0	3.32 (0.21)	356.7 (0.07)	29 (0.81)
3.0	3.33 (0.21)	310 (0.07)	28 (0.81)
7.0	2.89 (0.21)	207.8* (0.07)	26 (0.81)
46.5	2.17* (0.24)	81.4* (0.07)	25* (0.92)
165.0	–	–	–
Zinc			
Control	3.30 (0.21)	295.6 (0.06)	29 (0.81)
< 3.0	3.59* (0.21)	403.3* (0.06)	30 (0.81)
4.0	3.40 (0.21)	367.8 (0.06)	30 (0.81)
10.6	3.86* (0.21)	420.0* (0.06)	30 (0.81)
62.0	3.63* (0.21)	364.4 (0.06)	30 (0.81)
165.0	–	–	–

*Indicates significantly different from controls.

– Represents no samples taken

The lowest observed effect concentrations (LOEC) for stage 25 tadpoles exposed to copper was 46.5 µg/L for survival and length (Tables 3, 4), and 7 µg/L for development and weight (Table 4). Environmental concentrations of copper documented near CLF breeding grounds (47.5 mg/L) were more than three orders of magnitude greater than the LOECs for copper, based on mortality, development and length. The LOEC of 7 µg/L copper based on weight observed in the chronic study are considerably lower than the 96-hour LC50s of 60 µg/L reported for *Rana pipiens* (Lande and Guttman 1973) and 39 µg/L observed for *Rana hexadactyla* (Khangarot et al. 1985). Most of the mortality in the 60 day chronic study occurred within the first week of exposure, thus it is possible that mortality could occur as a result of episodic exposure to copper from rain water runoff.

In contrast to cadmium and copper, zinc was not toxic to CLF tadpoles at the highest concentration tested (165 µg/L) during the 60-day exposure (Table 3). Exposure to zinc appeared to have a stimulatory effect on growth and development compared to unexposed controls (Table 4). A zinc LC50 of 2.1 mg/L was reported for *Rana hexadactyla* tadpoles (Khangarot et al. 1985), and most published LC50s for other amphibian tadpoles are greater than 19 mg/L (Linder and Grillitsch 2000).

Copper was especially toxic causing both mortality and reduced growth of CLF tadpoles at concentrations potentially found in CLF habitat. Cadmium was not lethal in chronic exposures, but reduced growth was observed at levels above environmental concentrations. Zinc did not prove to be lethal, but instead had a stimulatory effect on growth. We determined the onset of toxicity occurs within a few days of exposure, thus pulsed exposures from rain events could potentially be acutely toxic to tadpoles of this species.
